# Mania and alzheimer disease, review and case report

**DOI:** 10.1192/j.eurpsy.2024.1323

**Published:** 2024-08-27

**Authors:** M. Garcia Moreno, A. De Cos Milas, L. Beatobe Carreño, P. Del Sol Calderón, A. Izquierdo de la Puente

**Affiliations:** ^1^Psychiatry, Hospital Universitario Puerta de Hierro Majadahonda; ^2^Psychiatry, Hospital Universitario de Móstoles, Madrid, Spain

## Abstract

**Introduction:**

There are numerous organic causes that can be related to affective symptoms such as neurological, metabolic, infectious and pharmacological. Neurological conditions associated to affective symptoms include vascular lesions, tumors, infections, seizures and dementia. Within cognitive impairment conditions, depressive symptoms are more frequent in vascular dementia and Alzheimer disease, and behavioral or manic symptoms in frontotemporal dementia although we cannot rule out less common associations.

**Objectives:**

To review about organic mania due to dementia

**Methods:**

We carry out a literature review about organic mania accompanied by a clinical description of one patient with manic symptoms and cognitive impairment.

**Results:**

A 80-year-old male was admitted to the short-term hospitalization unit from the emergency department due to maniform symptoms. He had believed for weeks that he was millionaire and capable to cure all the diseases in the world, reason for which he had given away many of his belongings and had tried to register the patent for his invent. He also had future plans to invest all the money he earned from the patent in the construction of roads in Latin America. He had not previous history of mental illness. Neurological study concluded a diagnosis of Alzheimer disease. It was treated as a manic episode with a mood stabilizer and antipsychotic, with partial resolution of the condition.

**Conclusions:**

It is common to find depressive symptoms in cognitive disorders. Although manic symptoms are much more frequent in frontotemporal dementia or other organic disorders, we can also find them in patients with Alzheimer disease. Since there is no specific curative treatment for this disease, concomitant psychopharmacological treatment is recommended if manic symptoms appear.

**Disclosure of Interest:**

None Declared

